# What remains after the money ends? Evidence on whether admission reductions continued following the largest health and social care integration programme in England

**DOI:** 10.1007/s10198-024-01676-0

**Published:** 2024-03-09

**Authors:** Vasudha Wattal, Katherine Checkland, Matt Sutton, Marcello Morciano

**Affiliations:** 1 Health Organisation, Policy and Economics (HOPE) Research Group, University of Manchester, Manchester, M13 9PL UK; 2 Department of Economics “Marco Biagi”, University of Modena and Reggio Emilia, Modena, Italy; 3 Visiting Research Associate, Care Policy and Evaluation Centre (CPEC), London School of Economics, London, UK

**Keywords:** Integrated care, England, Vanguard, New care models, Hospital admissions, D04, H51, I11, I18

## Abstract

We study the long-term effects on hospital activity of a three-year national integration programme. We use administrative data spanning from 24 months before to 22 months after the programme, to estimate the effect of programme discontinuation using difference-in-differences method. Our results show that after programme discontinuation, emergency admissions were slower to increase in Vanguard compared to non-Vanguard sites. These effects were heterogeneous across sites, with greater reductions in care home Vanguard sites and concentrated among the older population. Care home Vanguards showed significant reductions beginning early in the programme but falling away more rapidly after programme discontinuation. Moreover, there were greater reductions for sites performing poorly before the programme. Overall, this suggests the effects of the integration programme might have been lagged but transitory, and more reliant on continued programme support.

## Introduction

Pilot policy experiments are often natural predecessors of large-scale implementation [1]. The “success” of pilot experiments generally involve complex judgments over evolving policy objectives [2], with effects that are often not immediate and may take time to emerge. This is especially true if there is a learning period following implementation or lags in full implementation [3, 4]. However, the question of whether the beneficial impacts (if any) of a pilot experiment could persist after its discontinuation has received limited attention in policy evaluation literature. Undertaking impact evaluation, well beyond programme duration, can be informative about underlying mechanisms and circumstances that lead to permanent changes [5, 6].

We examine this in the context of integrated health care programmes. Health care systems around the world are being re-designed with a focus on delivering care in a resource efficient manner while ensuring timely and quality care to the patient. The focus within high-income countries is upon elderly population and/or those with complex health needs, whereas low-income countries are gradually moving towards addressing emerging challenges of dual (communicable and non-communicable) disease burden [7]. Among high-income countries, several previous integration programmes have remained localised to facilitate significant change at grassroot levels [8]. However, there are also working models of system wide integration efforts [9].

In this paper, we undertake impact evaluation for the Vanguard integrated health and care programme in England. This pilot programme included a mix of models that targeted a ‘high-risk’ group as well as broad population-based approaches. It was aimed at delivering care through an integrated system developed via enhanced coordination between general practitioners, communities, hospitals and social care services.

This programme is relevant for at least three reasons. Firstly, for its scale: it was a flagship National Health Service (NHS) England programme, running from 2015 to 2018, costing about $\pounds$389 million and covering a population of around 5 million (around 9% of the entire population in England). It was congruent to some extent with previous integrated care programmes piloted in England in terms of target population-older and those with complex conditions [10]. Second, for its scope: it was aimed at developing new models of care that would be sustainable within and beyond the Vanguards [11]. Third, for its policy relevance: The NHS Long-Term Plan [12, p. 13] announced the commitment to spread the innovative practices piloted with the Vanguard initiative across England.

In the initial stages, the Vanguards were allowed to set their own objectives with some guidelines from NHS England. But by the final year, the funding of the sites were linked to demonstration of reduction in emergency admissions and hospital bed days [13]. Previously, Morciano et al. [14] documented how the Vanguard programme slowed the persistent rise in hospital emergency admissions observed in England [15] during the programme period. However, the overall modest net reductions in the emergency hospital admissions of Vanguard sites largely occurred in the final year of the programme. They were also heterogeneous across initiatives and among sites differently exposed to previous integration initiatives [16]. However, legacy effects of the programme are not yet known.

In the field of medicine, legacy effects of a therapy are treatment effects that persist or emerge some time after treatment ends [17]. In a narrative review, Folz and Laiteerapong [17] show that the duration of follow-up period to be examined can vary from 2–5 years to until decades after. There are examples from other fields such as public economics wherein, Roper and Hewitt-Dundas [18] examined the legacy effects of public subsidies on private innovation 4–6 years after the initial subsidy. In the case of policy experiments, we examine whether changes adopted during the Vanguard initiatives were integrated into general capabilities of the institution, and therefore evaluate legacy effects.

This paper builds on Morciano et al. [14] in two ways. First, we extended the period of analysis to assess whether the beneficial effects of the Vanguard programme persisted after the programme finished using a standard difference-in-differences setting. Our follow-up period spans from the end of the Vanguard programme to the start of the COVID-19 pandemic. Doing so we are able to distinguish between short-term effects (during the treatment period itself) and long-term effects of the programme (post-programme discontinuation). Second, we use conditional quantile regressions to assess whether the programme led to heterogeneous outcomes among treated sites during and after the programme compared to the levels observed in the pre-Vanguard period for untreated sites.

Theoretically, the effect on outcomes post-programme discontinuation may be ambiguous. Any sustainable organisational, managerial and/or technological changes made during the programme period might lead to persistent effects on outcomes.^1^ But on the other hand, the support Vanguard sites received may have been pivotal to generating the beneficial effects seen at the time. Therefore, the effects may not persist without continued funding. Further, a stability (slow down) in net outcomes might also lead to an appearance of convergence (divergence) of trends between Vanguards and non-Vanguard sites. However, such convergence itself may come from well-performing or poor-performing sites. From a policymaker’s perspective, this insight is critical to knowing when to measure impact and when to discontinue investment.

Our difference-in-differences estimates show that after the end of the Vanguard programme average emergency admission rates were slower to increase among Vanguard relative to non-Vanguard sites. Furthermore, we find that the net reductions were greater at the upper end of the distribution (i.e., for sites with high admission rates). However, the net reduction in the post-Vanguard period became smaller and non-significant towards the end of the period we have covered, suggesting that the effects were lagged rather than permanent.

## Vanguard programme: details

The genesis of the Vanguard programme came about in 2014 in the NHS England’s Five Year Forward View (FYFV) which recognised that instead of structural reform involving a ‘one size fits all’ model, new ways of working may need to be developed to improve care delivery [21]. Thus, the core objective of the Vanguard programme was to create integrated systems that join up different arms of health and care services through innovative models. There were two ‘population-based’ Vanguard schemes (Multi-speciality Community Providers (MCPs) and Primary and Acute Care Systems (PACS)). Population-based vanguards were aimed at moving specialist care for the general population out of hospitals and into the community by fostering closer collaboration between GPs, hospitals, communities and social care services. There was also the ‘care home’ Vanguard scheme (The Enhanced Care in Care Homes (ECH)) aimed at improving the quality and coordination of health, care and rehabilitation services for care home residents by increasing the medical support available and by promoting collaboration between the NHS, local authorities, the voluntary sector, carers and relatives [22, 23]. These were aimed at delivering integrated care in the community involving primary, secondary, social and community care. There were other Vanguards focused on improving coordination among hospitals and emergency services as well.^2^ In all, 50 local areas were selected to act as Vanguards for the five proposed models. Subsequently, a support programme was devised to help develop and spread these new models of care within and beyond the Vanguards, which included a national lead for each model, support to develop logic models for local schemes, local account managers, learning and networking events, etc [16, 24]. The Vanguard programme also received substantial funding to support service changes within eligible sites. The total costs estimated by NAO include, direct costs at $\pounds$329 million and another $\pounds$60 million for national support and monitoring [25].

## Data and descriptives

We received data from NHS England on monthly counts of emergency admissions from 01 April 2013 until just ahead of the pandemic, 01 January 2020.^3^ The time horizon spans over 24 months before the introduction of the Vanguard programme, 36 months of the programme and 22 months after its termination. We focus on two ‘population-based’ Vanguard schemes as well as ‘care home’ Vanguard scheme.

As in Morciano et al. [14], the analysis is aggregated at site level. A treated site is defined as a set of practices within a Clinical Commissioning Group (CCG) that were exposed to the Vanguard programme. All practices in a CCG not exposed to the programme or a part of a CCG where some practices are not exposed to the programme, are classified as control sites.^4^ We therefore observe 24 sites involved in ‘population-based’ models (PACS and MCP combined), five sites exposed to ‘care home’ (ECH), and 175 not exposed sites that form our control group.^5^ Accordingly, our sample comprises 16,728 observations.

We measure hospital activity through Emergency Admissions (EA) which are those with a ‘specific acute’ treatment function code. Better integrated care in the community might plausibly affect (preventable) emergency route into hospital, less plausibly elective admissions. To account for different population sizes, we analysed EA rate per 1000 persons.

**Fig. 1:**
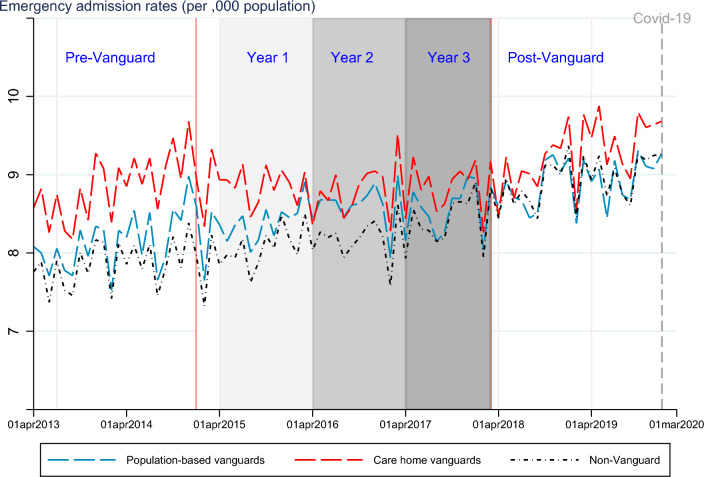
Trends in emergency admission rates for Vanguard and non-Vanguard sites

In Fig. 1, we report a time series plot of monthly EA rates observed in the treated and control groups. Emergency admissions were higher for the treated sites in the pre-intervention period. The population-based (PACS/MCP) sites follow a similar pre-intervention trend to the control groups, except just before the call for expressions of interest in the Vanguard programme was issued (November–December 2014). Emergency admission rates in the care home sites rose faster than the control sites just before the Vanguard programme started. However, we will later show in “Results”, through various parallel trends checks, that the overall pre-Vanguard trends across treated and control group were similar.

In line with what has already been reported [14], Fig. 1 shows Vanguard initiative slowed the rise in EA rates observed in England during the programme period in the treated groups, especially for care home Vanguard sites, closing the initial gap in EA rates with the non-Vanguard sites. After programme discontinuation, EA rates for care home Vanguard sites rose again, with the re-emergence of the initial gap. On the other hand, for population-based sites the converging trends which emerged in year 3 persisted in the post-Vanguard period.

One way to assess convergence in EA rates, evidenced by a reduction in dispersion or compression in the EA rates distribution over time, is by looking at trends in the 25th and 75th percentiles of logged EA rates by groups.^6^

**Fig. 2:**
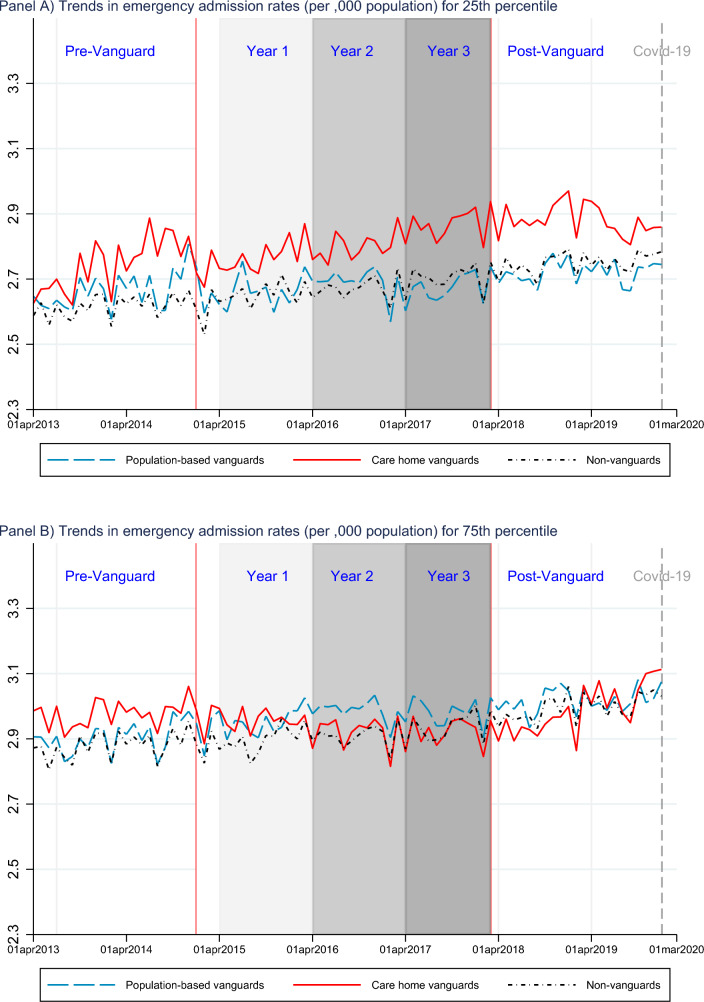
Trends in emergency admission rates (per ,000 population) for Vanguard types, by moments of the log-distribution

Among better performers (25th percentile, Fig. 2, panel A), an upward trend is found for both the treated and the control groups throughout the programme duration, which continues after its end. A slowdown in the rising trend is observed for care home Vanguards in the first year of the programme and after its termination. The better performing population-based sites had lower EA rates than non-Vanguards from the third year of the programme and after its end.

Poorest performing (75th percentile, Fig. 2, panel B) control sites also experienced rising trends. In comparison, care home Vanguards experienced a steady reduction in EA rates throughout the three years of the programme, before rising again after its end. Poorly performing population-based Vanguards experienced a slight increase in EA rates at the start of the programme, followed by a reduction around the final year and continues to remain stable for most of the post-Vanguard period.

These graphical representations indicate that convergence in EA rates emerges due to reductions in the poorest performing Vanguard sites. Regression analysis in the following section sheds further light upon these trends.

## Empirical specification

To examine the net impact of Vanguard on hospital activity, we employ a two-way (site and month) fixed-effect OLS estimator in a difference-in-differences setup, using the following specification:

1 $$\begin{aligned} \begin{aligned} ln(Y_{it}) = \alpha _{i}+\beta _{t}+ \sum _{j}\gamma _{j}V_{i}+ \sum _{k}\delta _{k}P_{it}+ \sum _{j}\sum _{k}\eta _{jk}V_{i}*P_{t} +\theta X_{it} + \epsilon _{it} \end{aligned} \end{aligned}$$

$ln(Y_{it})$ identifies the logged outcome of interest (EA rates) for site *i* and month *t*. $\alpha$ and $\beta$ identify the site and month fixed effects, respectively. To account for factors that vary over time within site, we add controls (*X*) for site-level population structure as the monthly proportion of population by age-groups (0–24; 25–64; 65 and older). *V* identifies three groups: the control group of non-Vanguard sites ($j = 0$) and the two treated groups of sites exposed to population-based ($j = 1$) and care home ($j = 2$) Vanguards. *P* identifies programme timing in quarters: the pre-Vanguard period ($k<0$), quarter 0 to quarter 12 of the Vanguard programme ($k=0,\ldots ,12$) and the post-Vanguard period ($k=13,\ldots ,18$). The key parameters of interest are those associated with the interaction terms $V_{i}$ and $P_{t}$, $\eta _{jk}$. Specifically, they measure the net change among population-based Vanguards in each quarter k ($\eta _{1k}$) of the programme and in the follow-up period compared to non-Vanguard sites ($j = 0$), compared to the gap between them in the pre-Vanguard period ($k<0$). Similarly, the net change among care home Vanguards is captured by $\eta _{2k}$ for each quarter *k*.

A focus on the mean net impact of the programme may mask meaningful heterogeneous treated sites’ responses to the programme. We therefore present a model for conditional quantile regressions to estimate the effect of programme status on emergency admissions distribution. There are several merits to doing this. Firstly, the conditional mean is more prone to distorting effects of outliers, to which conditional quantiles are more robust. Secondly, conditional quantiles provide more valuable information about the full distributional impact of the programme.

Our approach is based on Machado and Silva [26]. Their model allows for additive fixed effects and multiple treatment groups both of which are relevant for our set up. As before, the treatment variable ($V_{i}$) refers to population-based or care home Vanguard sites versus a non-Vanguard site. The $\tau$-quantile distribution of our outcome of interest $-ln(Y_{it})$, $Q_{ln(Y_{it})}$ is defined as:

2 $$\begin{aligned} \begin{aligned} Q_{ln(Y_{it})}(\tau | V_{i}, X_{it}) =(\alpha _{i} +\delta _{i}q(\tau )) + V^{\prime }_{i}\beta _{1} + X^{\prime }_{it}\beta _{2} + W^{\prime }_{t}\beta _{3}+ Z^{\prime }_{it}\gamma q(\tau ) \end{aligned} \end{aligned}$$

In the present paper, $X_{it}$ includes time varying variables such as the proportion of population by age group and the interaction terms of Vanguard type and treatment period. $Q_{ln(Y_{it})}(\tau |X_{it})$ is the quantile distribution of logged emergency admissions conditional on the location of $X_{it}$. Whereas $W_{t}$ indicates time fixed effects. *Z* is a k-vector of known differentiable transformations of *X* with element *l*, $Z_{l}=Z_{l}(X)$. $(\alpha _{i} +\delta _{i}q(\tau ))$ is the scalar coefficient that provides an estimate of the fixed effect at quantile $\tau$ for a given unit *i*. This represents the effects of time invariant unit specific characteristics which have variable impacts across different regions of the conditional distribution of outcome variable [26]. Accordingly, $\alpha _{i}$ can be interpreted as the average effect for unit *i*. We use robust standard errors, and we did not cluster based on Abadie et al. and Roth et al. [27, 28]. We explain this further in “Appendix 3: Clustering standard errors”.

To infer about distributional effects of Vanguard programme to individual sites, we need to be able to assume rank preservation, i.e. the ranks of outcomes are same across treatment states. In the present context, this implies that better performing sites in the pre-Vanguard period, remain better performers having been selected into the programme. A less strict assumption that is often made in the literature on quantile treatment effects is one of rank similarity, which requires that there are no systematic deviations between distribution of outcomes across treatment states.^7^ In the presence of rank preservation/similarity, quantile regressions would inform us of the following; (a) whether observed convergence is due to those at the upper or lower end of the distribution; (b) whether the effect of the programme varies across the distribution of the outcome variable. However, there are conditions under which rank similarity may not always hold.^8^ Moreover, this is not always testable if systematic deviations may be caused by unobservables. In the absence of rank preservation, we can still make meaningful inferences about the effect of the programme on the overall distribution of the outcome variable [31].

## Results

**Fig. 3:**
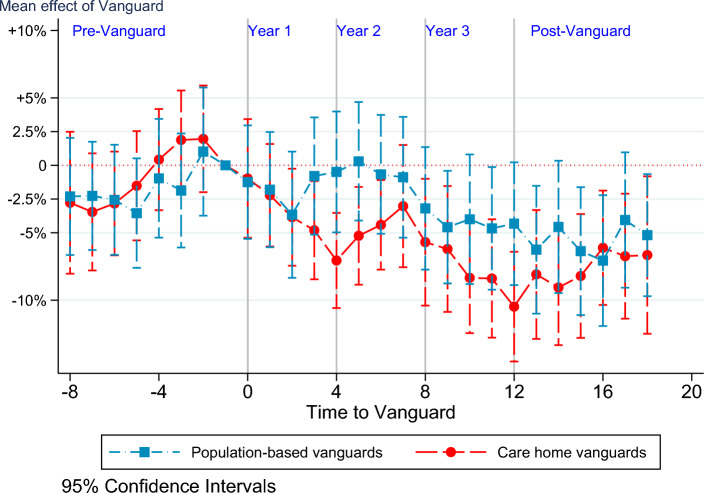
Mean effect at various stages of Vanguard programme. The points correspond to the coefficient for each Vanguard type relative to the baseline non-Vanguards and the *x*-axis denotes time in quarters

**Fig. 4:**
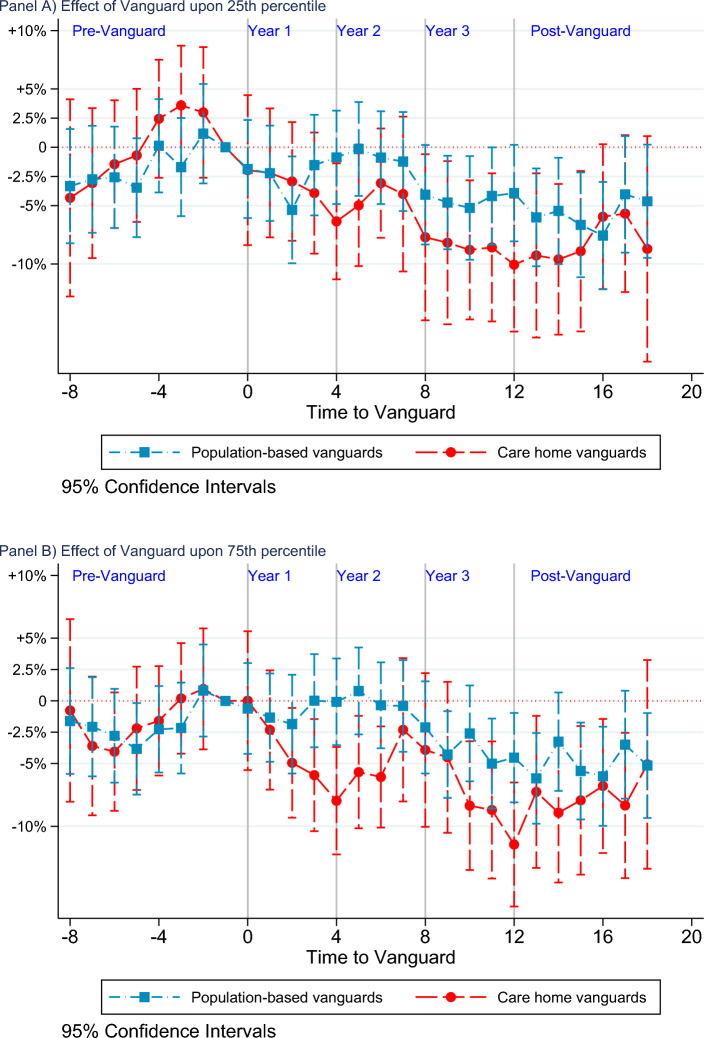
Quantile effect of Vanguard programme upon emergency admission rates. Each point corresponds to the coefficients for each Vanguard type relative to the baseline non-Vanguards and the *x*-axis denotes time in quarters. Vertical lines identify 95% pointwise confidence intervals

For simplified presentation, estimates are reported by quarters from/to programme’s start (quarter 0) in an event study format in Figs. 3 and 4. This representation allows the common trend assumption to be easily checked for quarters $<0$. Moreover, it helps in detecting how the net impact of the programme evolves over time among treated sites versus the control group. It is evident from Fig. 3 that the parallel trend assumption holds in pre-Vanguard period for each Vanguard group and for all periods.^9^

For the care home Vanguard group, there was a significant net decline in emergency admission rates from the later quarters of year 1, which persisted in year 2 (except quarter 7) and further declined in year 3. In the post-Vanguard period, the decrease appears to slow down. A net impact among population-based sites emerged only in year 3 (except quarters 10 and 12) and remained significant for most of the post-Vanguard period (except quarters 14 and 17).^10^

Table 1 reports results from the quantile regressions for the 10th, 25th, 50th, 75th and 90th quantiles estimated using Eq. 2. For population-based sites, at the end of year 3 (11th and 12th quarter), there appear heterogenous effects of increasing magnitude at higher quantiles, with the trend reversing/remaining stable in the post-Vanguard period (quarters 13, 15, 16, 18). Similarly for care home Vanguards, in the initial years the net effects are higher when moving to higher quantiles, but in the third year the differences across quantiles are stabilised or reversed. Towards the end of the post-Vanguard period, the increasing net impact across quantiles appeared again.

These results suggest that the effect of the programme is heterogenous with the effect being greater for poor-performing sites (for most of the programme duration). For care home vanguards, the net reduction in emergency admissions emerged mainly from early improvements in poor-performing sites. Under the assumption of rank preservation this would imply that the Vanguard programme had a greater positive effect upon pre-existing poorer performers than those that were already performing well in the pre-Vanguard period.

**Table 1:** Panel quantile results

	Quantile				
	0.1	0.25	0.5	0.75	0.9
*Quarter* $\times$ *Vanguard* ($j=1$)					
− 8 $\times$ Population-based vanguards	− 0.041	− 0.033	− 0.024	− 0.016	− 0.010
− 7 $\times$ Population-based vanguards	− 0.030	− 0.027	− 0.024	− 0.021	− 0.018
− 6 $\times$ Population-based vanguards	− 0.025	− 0.026	− 0.027*	− 0.028	− 0.029
− 5 $\times$ Population-based vanguards	− 0.033	− 0.035	− 0.037**	− 0.038**	− 0.040
− 4 $\times$ Population-based vanguards	0.012	0.001	− 0.011	− 0.023	− 0.032
− 3 $\times$ Population-based vanguards	− 0.015	− 0.017	− 0.019	− 0.022	− 0.023
− 2 $\times$ Population-based vanguards	0.013	0.012	0.010	0.008	0.007
0 $\times$ Population-based vanguards	− 0.024	− 0.019	− 0.012	− 0.006	− 0.001
1 $\times$ Population-based vanguards	− 0.026	− 0.022	− 0.018	− 0.013	− 0.010
2 $\times$ Population-based vanguards	− 0.069**	− 0.054**	− 0.035**	− 0.019	− 0.005
3 $\times$ Population-based vanguards	− 0.022	− 0.015	− 0.007	0.000	0.006
4 $\times$ Population-based vanguards	− 0.012	− 0.009	− 0.004	− 0.001	0.002
5 $\times$ Population-based vanguards	− 0.006	− 0.001	0.003	0.008	0.011
6 $\times$ Population-based vanguards	− 0.011	− 0.009	− 0.006	− 0.004	− 0.002
7 $\times$ Population-based vanguards	− 0.016	− 0.012	− 0.008	− 0.004	− 0.001
8 $\times$ Population-based vanguards	− 0.049	− 0.041*	− 0.030**	− 0.021	− 0.014
9 $\times$ Population-based vanguards	− 0.049*	− 0.047**	− 0.045***	− 0.043**	− 0.041*
10 $\times$ Population-based vanguards	− 0.064*	− 0.052**	− 0.038**	− 0.026	− 0.016
11 $\times$ Population-based vanguards	− 0.038	− 0.042**	− 0.046***	− 0.050***	− 0.053**
12 $\times$ Population-based vanguards	− 0.037	− 0.039*	− 0.042***	− 0.045**	− 0.047*
13 $\times$ Population-based vanguards	− 0.059*	− 0.060***	− 0.061***	− 0.062***	− 0.062**
14 $\times$ Population-based vanguards	− 0.064*	− 0.055**	− 0.043***	− 0.033	− 0.024
15 $\times$ Population-based vanguards	− 0.071**	− 0.067***	− 0.061***	− 0.056***	− 0.052*
16 $\times$ Population-based vanguards	− 0.083**	− 0.076***	− 0.067***	− 0.060***	− 0.054*
17 $\times$ Population-based vanguards	− 0.043	− 0.040	− 0.037**	− 0.035	− 0.033
18 $\times$ Population-based vanguards	− 0.044	− 0.046*	− 0.049***	− 0.052**	− 0.053*
*Quarter* $\times$ *Vanguard* ($j=2$)					
− 8 $\times$ Care home vanguards	− 0.059	− 0.043	− 0.024	− 0.008	0.006
− 7 $\times$ Care home vanguards	− 0.028	− 0.031	− 0.033	− 0.036	− 0.038
− 6 $\times$ Care home vanguards	− 0.003	− 0.014	− 0.028	− 0.040*	− 0.050
− 5 $\times$ Care home vanguards	− 0.000	− 0.007	− 0.015	− 0.022	− 0.027
− 4 $\times$ Care home vanguards	0.043	0.024	0.003	− 0.016	− 0.031
− 3 $\times$ Care home vanguards	0.051	0.036	0.018	0.002	− 0.011
− 2 $\times$ Care home vanguards	0.039	0.030	0.019	0.010	0.002
0 $\times$ Care home vanguards	− 0.028	− 0.020	− 0.009	0.000	0.007
1 $\times$ Care home vanguards	− 0.021	− 0.022	− 0.023	− 0.023	− 0.024
2 $\times$ Care home vanguards	− 0.020	− 0.029	− 0.040**	− 0.049**	− 0.057*
3 $\times$ Care home vanguards	− 0.030	− 0.039	− 0.050***	− 0.059***	− 0.067**
4 $\times$ Care home vanguards	− 0.056	− 0.064**	− 0.072***	− 0.080***	− 0.086***
5 $\times$ Care home vanguards	− 0.047	− 0.050*	− 0.053***	− 0.057**	− 0.059*
6 $\times$ Care home vanguards	− 0.017	− 0.031	− 0.047***	− 0.061***	− 0.072**
7 $\times$ Care home vanguards	− 0.048	− 0.040	− 0.031	− 0.023	− 0.017
8 $\times$ Care home vanguards	− 0.094*	− 0.077**	− 0.057**	− 0.039	− 0.025
9 $\times$ Care home vanguards	− 0.098*	− 0.082**	− 0.062**	− 0.045	− 0.031
10 $\times$ Care home vanguards	− 0.090**	− 0.088***	− 0.086***	− 0.084***	− 0.082**
11 $\times$ Care home vanguards	− 0.085*	− 0.086***	− 0.086***	− 0.087***	− 0.087**
12 $\times$ Care home vanguards	− 0.094**	− 0.101***	− 0.108***	− 0.115***	− 0.120***
13 $\times$ Care home vanguards	− 0.102*	− 0.093***	− 0.082***	− 0.073**	− 0.065
14 $\times$ Care home vanguards	− 0.099**	− 0.096***	− 0.092***	− 0.089***	− 0.087**
15 $\times$ Care home vanguards	− 0.093*	− 0.089**	− 0.084***	− 0.079***	− 0.076*
16 $\times$ Care home vanguards	− 0.056	− 0.059*	− 0.064***	− 0.068**	− 0.071*
17 $\times$ Care home vanguards	− 0.045	− 0.057*	− 0.071***	− 0.083***	− 0.093**
18 $\times$ Care home vanguards	− 0.103	− 0.087*	− 0.068**	− 0.051	− 0.037

*$$p<0.10$$, **$$p< 0.015$$, ***$$p< 0.01$$

Figure 4 reports on graphical presentation of estimates for the 25th (panel A) and 75th (panel B) percentiles from Table 1. At the 25th percentile, we found no significant differences among treated sites in the pre-Vanguard period. Further, we observe no significant impact of population-based or care home Vanguard initiatives for the first two years of the programme (except a transient significant effect in quarter 2 of year 1 for population-based Vanguards and quarter 4 for care home Vanguards). Significant net impacts emerged from the beginning of year 3 and remained significant at 95% level up to three quarters of the post-Vanguard period.

At the 75th percentile, pre-Vanguard parallelism holds for care home Vanguards and for population-based Vanguards with the exception of the 5th quarter in the pre-Vanguard period. We found a significant net reduction in EA rates for care home vanguards that started around the mid of year 1 and remained more or less so^11^ for the entire programme duration. On the other hand, population-based sites register a net reduction for most of year 3, with a lagged effect that continues for most of the post-Vanguard period.^12^

We do an additional sensitivity check to establish how the programme affected sites that were better/poor performers before the start of the programme. This was done by assigning site membership into quantiles based on monthly values of emergency admissions from April 2013-March 2014 (first year of pre-vanguard period). Given the transient movement across quantiles, we focused on sites that were consistently in the 25th (75th) quantile for at least 6 months (50%) in that year.^13^ The dependent variable was computed by taking the difference in the mean observed log of emergency admission rates at a point of time beginning April 2014 until January 2020, between sites that were pre-determined to be in the top and bottom quartiles. The estimation was carried out using a feasible generalised least squares method and imposing an error structure with heteroskedastic panels.

The results from quantile regressions (Table 1) slightly differ in magnitude (but the statistical significance of the parameters of interest diluted significantly) from the estimates obtained when quantile membership is pre-determined according pre-vanguard EA rates (results available upon request). There are two main reasons for this. First, there are methodological differences. Computing average treatment effects for pre-determined quantiles through ordinary least squares involves minimising sum of squared residuals, whereas quantile regression computes estimates by minimising the sum of absolute residuals. Second, since assigning membership on pre-vanguard data severely restricts our sample (6 population-based sites and 2 care home sites), thereby leading to small sample/aggregation bias seen in “Appendix 1: Testing aggregation bias”. For this reason, we preferred to draw our inferences from the analysis of disaggregated data.

## Age group analysis

**Fig. 5:**
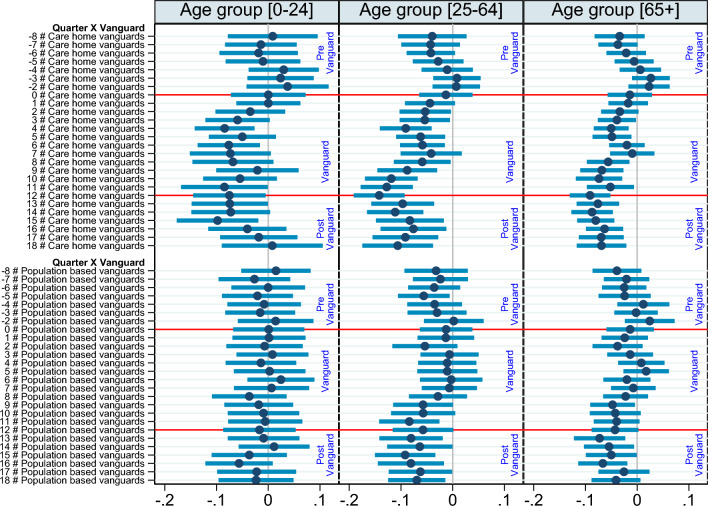
Mean effect at various stages of Vanguard programme across three age groups. The dependent variable is log of emergency admissions per 1000 population in a given age group. The points correspond to the coefficient for each vanguard type relative to the baseline non-vanguards. The confidence bands are at 95% level

We next present differences of policy effects by age group. We do this by estimating our original difference-in-differences regression as per Eq. 1 with the dependent variable as total EA rates per 1000 persons in each of the three age groups. We control for site level-population structure in other age groups. The results are presented in Fig. 5.

These results show that among the youngest age group (0–25), the effect of care home Vanguards upon emergency admissions reduction is significant for a few quarters (4, 6, 11, 12) during the programme and to a limited extent in the post-Vanguard period (13 and 15 quarters). However, there are no significant effects of the population-based vanguards on emergency admissions amongst the youngest cohort. In case of the oldest population group, significant effects of care home Vanguard on emergency admissions are evident early in the programme (from quarter 2). While the effects in the 6th and 7th quarters are not significant, there is a downward trend afterwards until the end of the programme. After programme discontinuation, the reductions in emergency admissions tend to remain significant and fade only slightly in the last three quarters. In contrast, the effect of population-based vanguards among the oldest cohort is not so clear. During the programme the effects appear insignificant (except quarter 9). Though in the post Vanguard period, the lower admissions rates appear to persist until the last two quarters. For the adult age cohort (25–64), the effects of care home Vanguard on emergency admissions appear similar to the oldest group in the early stages of the programme, with a clear downward trend after the 7th quarter. In the post-programme period, the effects remain significant and the EA rates appears to become slightly higher but remain below pre-Vanguard levels. For population-based vanguards, significant reductions in emergency admissions for adults appear only at the end of the programme (quarters 9 and 11) and persist at the same levels in the post Vanguard period. Detailed regression output is in Table 2.

Overall, our results suggest that persistent effects come from care home Vanguards benefiting the oldest cohorts the most. This might explain why the average effect on care homes in the post Vanguard period appears to disappear more rapidly (in Fig. 1), since it is likely that the reductions for the older cohorts were muted by sharper increases in EA rates among the younger cohort. This also supports our understanding that care homes have more older residents and/or those more vulnerable to emergency admissions, and thus the targeted nature of interventions produce effects early on and may persist for longer for this group.

**Table 2:** Difference-in-differences estimates by age group

	Age groups		
	[0–24]	[25–64]	[65+]
*Quarter* $\times$ *Vanguard* ($j=1$)			
− 8 $\times$ Population-based vanguards	0.015	− 0.032	− 0.039
− 7 $\times$ Population-based vanguards	− 0.027	− 0.024	− 0.021
− 6 $\times$ Population-based vanguards	− 0.000	− 0.036	− 0.025
− 5 $\times$ Population-based vanguards	− 0.021	− 0.056**	− 0.024
− 4 $\times$ Population-based vanguards	− 0.008	− 0.035	0.012
− 3 $\times$ Population-based vanguards	− 0.016	− 0.030	− 0.002
− 2 $\times$ Population-based vanguards	0.014	0.002	0.024
0 $\times$ Population-based vanguards	0.001	− 0.013	− 0.014
1 $\times$ Population-based vanguards	0.002	− 0.013	− 0.024
2 $\times$ Population-based vanguards	− 0.007	− 0.054*	− 0.038
3 $\times$ Population-based vanguards	0.008	− 0.006	− 0.014
4 $\times$ Population-based vanguards	− 0.014	− 0.011	0.008
5 $\times$ Population-based vanguards	0.003	− 0.010	0.017
6 $\times$ Population-based vanguards	0.025	− 0.003	− 0.020
7 $\times$ Population-based vanguards	0.006	− 0.006	− 0.007
8 $\times$ Population-based vanguards	− 0.037	− 0.028	− 0.022
9 $\times$ Population-based vanguards	− 0.018	− 0.057*	− 0.048**
10 $\times$ Population-based vanguards	− 0.009	− 0.057*	− 0.042*
11 $\times$ Population-based vanguards	− 0.006	− 0.084***	− 0.040*
12 $\times$ Population-based vanguards	− 0.017	− 0.057*	− 0.043*
13 $\times$ Population-based vanguards	− 0.009	− 0.080**	− 0.073***
14 $\times$ Population-based vanguards	0.011	− 0.064**	− 0.054**
15 $\times$ Population-based vanguards	− 0.037	− 0.092***	− 0.050**
16 $\times$ Population-based vanguards	− 0.056*	− 0.081**	− 0.066***
17 $\times$ Population-based vanguards	− 0.022	− 0.062**	− 0.025
18 $\times$ Population-based vanguards	− 0.024	− 0.069**	− 0.041*
*Quarter* $\times$ *Vanguard* ($j=2$)			
− 8 $\times$ Care home vanguards	0.009	− 0.040	− 0.034
− 7 $\times$ Care home vanguards	− 0.014	− 0.043	− 0.037*
− 6 $\times$ Care home vanguards	− 0.019	− 0.042*	− 0.021
− 5 $\times$ Care home vanguards	− 0.010	− 0.028	− 0.006
− 4 $\times$ Care home vanguards	0.030	− 0.011	0.006
− 3 $\times$ Care home vanguards	0.024	0.008	0.027
− 2 $\times$ Care home vanguards	0.037	0.007	0.023
0 $\times$ Care home vanguards	0.000	− 0.013	− 0.014
1 $\times$ Care home vanguards	0.000	− 0.044*	− 0.017
2 $\times$ Care home vanguards	− 0.035	− 0.053**	− 0.033*
3 $\times$ Care home vanguards	− 0.059*	− 0.054**	− 0.039**
4 $\times$ Care home vanguards	− 0.084***	− 0.091***	− 0.050***
5 $\times$ Care home vanguards	− 0.050	− 0.062**	− 0.049**
6 $\times$ Care home vanguards	− 0.076**	− 0.058***	− 0.020
7 $\times$ Care home vanguards	− 0.073*	− 0.042	− 0.009
8 $\times$ Care home vanguards	− 0.068*	− 0.059**	− 0.056***
9 $\times$ Care home vanguards	− 0.021	− 0.088***	− 0.068***
10 $\times$ Care home vanguards	− 0.054	− 0.119***	− 0.074***
11 $\times$ Care home vanguards	− 0.085**	− 0.127***	− 0.052**
12 $\times$ Care home vanguards	− 0.075**	− 0.142***	− 0.091***
13 $\times$ Care home vanguards	− 0.074**	− 0.097***	− 0.076***
14 $\times$ Care home vanguards	− 0.072*	− 0.111***	− 0.087***
15 $\times$ Care home vanguards	− 0.098**	− 0.083**	− 0.080***
16 $\times$ Care home vanguards	− 0.040	− 0.076**	− 0.063***
17 $\times$ Care home vanguards	− 0.018	− 0.092***	− 0.069***
18 $\times$ Care home vanguards	0.008	− 0.106***	− 0.069***

This table shows difference-in-differences estimates of emergency admissions rates in the 22 months post-Vanguard for integrated care and care home initiatives compared to non-Vanguard sites and the pre-Vanguard period in each age group. Base period is the quarter immediately preceding the onset of the Vanguard period

*$$p<0.10$$, **$$p< 0.015$$, ***$$p< 0.01$$

## Limitations

There are a few limitations to this study worth noting. Our present results show the aggregate effect of integration upon emergency admissions without accounting for changes in case mix. Beyond reducing the number of hospital admissions, integration programmes may have led to less severe and/or shorter hospital days. While we do not have access to additional data on these aspects of emergency admissions, we expect that these factors would vary little over the study period. As such, these may be absorbed to some extent within area fixed effects and common time effects that we have included in our estimation.

Another limitation might come from confounding effects of other policies concomitant during the Vanguard period, implying that the legacy might not be from Vanguard alone. These effects to some extent may be captured in our econometric approach, since we include month fixed effects and if such policies targeted Vanguard sites, then they would be absorbed by site fixed effects. As an example of such a policy, we consider the Pioneer programme, aimed at promoting horizontal integration between health and social care systems [16], which ran from 2013 to 2018 (for details, see “Appendix 2: Effects of confounding policies”). We exploit the partial overlapping of the Pioneer programme with the Vanguard by running our main specification (1) using the sub-sample of sites that were not involved in the Pioneer programme. The results are confined in Table 4 in “Appendix 2: Effects of confounding policies”. We found that non-pioneer sites had detectable effects that continued after the programme termination, although the magnitude of the effects becomes smaller and becomes non-significant at the end of the sample period.

We report evidence of legacy effects for two years after the end of the programme, but before the onset of COVID-19 which is known to have altered activities especially in confined environments like care homes [33]. Whether initiatives such as Vanguard have helped institutions to be more resilient to pandemic is difficult to assess and beyond the scope of this paper.

Finally, we are also unable to specify the precise mechanism through which these legacy effects may be generated. This is because it is difficult to ascertain how funding was utilised to make the necessary changes due to the lack of financial accountability of spending patterns for the Vanguard initiatives [25].^14^

## Discussion

We examined the follow-up effects of Vanguard—an integrated care pilot programme that was active in England between 2015 and 2018. Since we report (in “Data and descriptives” section) higher emergency admissions among the treated sites in the pre-intervention period, it may then be expected, that with improved coordination in delivery of care, better care may reduce the likelihood of admissions within these sites. While confirming previous results in Morciano et al. [14], our expanded analysis reported lagged effects in the six quarters following the end of the programme. Care home sites are vulnerable to high levels of emergency admissions, given their residents are mainly older people [35, 36]. Therefore, focused interventions on a ‘high-risk’ population, living in confined environments such as care homes, are likely to produce detectable effects upon hospital activities quicker than wider population-based interventions. Our age group analysis supports this hypothesis.

Consistent with this, our analysis demonstrated that care home Vanguards showed significant reductions beginning early in the programme but falling away more rapidly after programme discontinuation. As care home sites catered to a more vulnerable category of the population, continued funding and integration support may have been critical to sustaining the effects seen during the programme. Moreover, care homes being smaller organisations might be less able to invest the integration funds towards making lasting changes, and so may have been more reliant on continued funding support.

Moreover, our analysis showed that for most of the programme, and to some extent in the post-Vanguard period, the reductions in hospital admission rates were greater in magnitude among poor-performing sites. These outcomes may have been influenced by the non-pecuniary support associated with the programme.^15^

Our results indicate that the programme has enabled reductions in hospital admission rates which have persisted beyond the programme period, supporting the thesis of legacy effects. More importantly, we found that the net reductions tended to fade away over time after the end of the programme and towards the end of the period we have covered. This suggests that integration efforts may have had lagged effects, but the effects were transitory.

Earlier evaluations have demonstrated that given the numerous hurdles in the integration process, it could take 5–6 years for any meaningful impacts of these efforts to show up [10]. Longer term engagements may allow enough time for stabilisation of processes. Our study has been able to demonstrate that some effects persist in the immediate follow-up period. From the perspective of policy evaluation, there may be a case for a longer term horizon to arrive at any clear conclusions about policy impact.

Finally, though the initial vision was to scale up all Vanguards to the national level, however this was implemented for only one of the models—ECH [12]. This is said to have been enabled as a direct result of Vanguard’s legacy in fostering strong relationships across service partner organisations [37]. But also since at the outset, populations in care homes are well-defined, homogeneous and services in this area were more underdeveloped to start with [14]. This indicates more effective population-based approaches to integrated care delivery may need to be explored again in the future.

## Figures and Tables

**Fig. 6:**
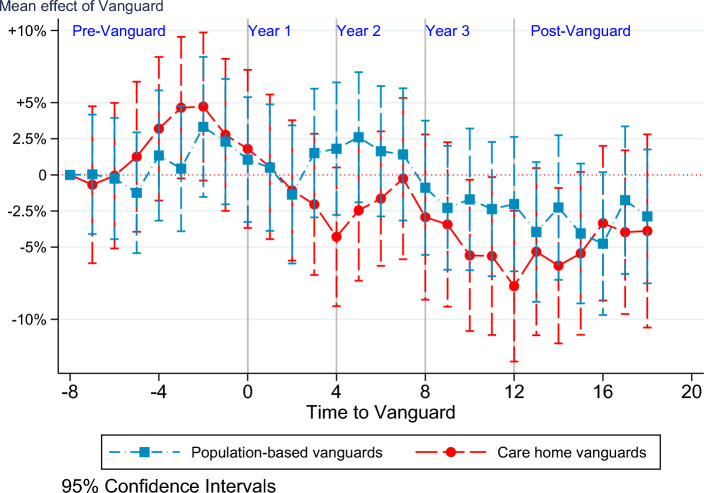
Mean effect at various stages of Vanguard programme with reference to the first quarter of the pre-Vanguard period. The points correspond to the coefficient for each Vanguard type relative to the baseline non-Vanguards and the *x*-axis denotes time in quarters

**Fig. 7:**
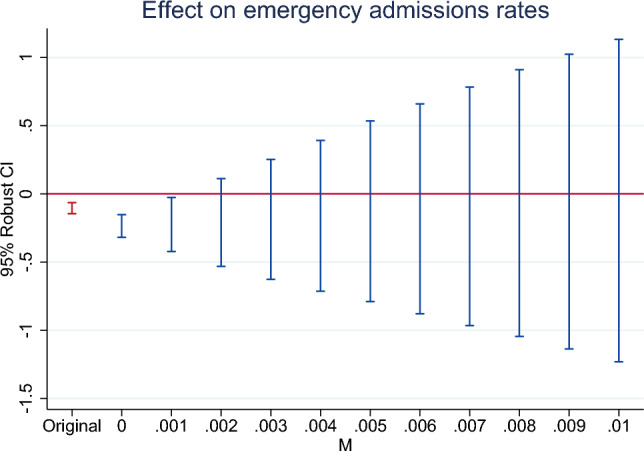
Sensitivity analysis for care home Vanguards at quarter = 12, which is the first post Vanguard period, using smoothness restrictions

## Data Availability

The data that support the findings of this study are not publicly available but can be requested through NHS England. Program codes are available upon request.

## Appendix 1: Testing aggregation bias

We checked for aggregation bias by comparing the overall results from Eq. 1 with those obtained from group level estimates. The latter involves obtaining mean values of emergency admissions for $V_{j}$ groups at each point of time. Since there are 3 groups; 2 treatment and 1 control group spanned over 81 months, we have 243 observations. Given this method of aggregation we do not include time fixed effects in the estimations. With the short panel and relatively longer time series, we implemented a feasible generalised least squares estimation. Table 3 contrasts our main estimates displayed in the paper (column 1) with group-level estimates obtained with and without assuming potential heteroskedasticity across panels (columns 2 and 3).

We found that panel level heteroskedasticity assumptions (column 2 vs column 3) has no impact on our estimates of interest. Group-level analysis confirms the magnitude of findings reported in the paper that mean emergency admissions rates slowed down for care home Vanguards in early stages of the Vanguard programme, whereas a net reduction emerged mainly in the third and final year of the programme for population-based vanguards. The group-level analysis also confirms the presence of legacy effects and/or lagged effects after programme discontinuation. However, the information loss resulting from the aggregation might have impacted on the precision of estimated parameters and statistical significance. This may be linked with higher standard errors in the aggregated equation due to correlation of residuals across disaggregated regressions [38]. As such, we found that the Vanguard programme has little to no significant effect upon Vanguard types during the programme. In the post-Vanguard period, the slow down appears to continue for care home Vanguards although the magnitude of the effect becomes smaller. For population-based sites, we found a consistent net reduction over the post-Vanguard period which is estimated to be significant at quarters 13, 15 and 16.

**Table 3:** Difference-in-differences estimates

	Group		
	Overall	Panel (hetero)	Panel (no hetero)
*Quarter* $\times$ *Vanguard* ($j=1$)			
− 8 $\times$ Population-based vanguards	− 0.023	− 0.021	− 0.021
− 7 $\times$ Population-based vanguards	− 0.023	− 0.021	− 0.021
− 6 $\times$ Population-based vanguards	− 0.026	− 0.024	− 0.024
− 5 $\times$ Population-based vanguards	− 0.035*	− 0.034	− 0.034
− 4 $\times$ Population-based vanguards	− 0.010	− 0.009	− 0.009
− 3 $\times$ Population-based vanguards	− 0.019	− 0.018	− 0.018
− 2 $\times$ Population-based vanguards	0.010	0.011	0.011
0 $\times$ Population-based vanguards	− 0.012	− 0.012	− 0.012
1 $\times$ Population-based vanguards	− 0.018	− 0.018	− 0.018
2 $\times$ Population-based vanguards	− 0.037	− 0.036	− 0.036
3 $\times$ Population-based vanguards	− 0.008	− 0.008	− 0.008
4 $\times$ Population-based vanguards	− 0.005	− 0.005	− 0.005
5 $\times$ Population-based vanguards	0.003	0.004	0.004
6 $\times$ Population-based vanguards	− 0.007	− 0.005	− 0.005
7 $\times$ Population-based vanguards	− 0.009	− 0.007	− 0.007
8 $\times$ Population-based vanguards	− 0.032	− 0.030	− 0.030
9 $\times$ Population-based vanguards	− 0.046**	− 0.044	− 0.044
10 $\times$ Population-based vanguards	− 0.040	− 0.039	− 0.039
11 $\times$ Population-based vanguards	− 0.047**	− 0.045	− 0.045
12 $\times$ Population-based vanguards	− 0.043*	− 0.042	− 0.042
13 $\times$ Population-based vanguards	− 0.063***	− 0.060*	− 0.060*
14 $\times$ Population-based vanguards	− 0.046*	− 0.042	− 0.042
15 $\times$ Population-based vanguards	− 0.064***	− 0.061*	− 0.061*
16 $\times$ Population-based vanguards	− 0.071***	− 0.068**	− 0.068**
17 $\times$ Population-based vanguards	− 0.041	− 0.037	− 0.037
18 $\times$ Population-based vanguards	− 0.052**	− 0.049	− 0.049
*Quarter* $\times$ *Vanguard* ($j=2$)			
− 8 $\times$ Care home vanguards	− 0.028	− 0.024	− 0.024
− 7 $\times$ Care home vanguards	− 0.035	− 0.032	− 0.032
− 6 $\times$ Care home vanguards	− 0.028	− 0.025	− 0.025
− 5 $\times$ Care home vanguards	− 0.015	− 0.013	− 0.013
− 4 $\times$ Care home vanguards	0.004	0.004	0.004
− 3 $\times$ Care home vanguards	0.019	0.020	0.020
− 2 $\times$ Care home vanguards	0.020	0.020	0.020
0 $\times$ Care home vanguards	− 0.010	− 0.011	− 0.011
1 $\times$ Care home vanguards	− 0.022	− 0.023	− 0.023
2 $\times$ Care home vanguards	− 0.038**	− 0.039	− 0.039
3 $\times$ Care home vanguards	− 0.048***	− 0.048	− 0.048
4 $\times$ Care home vanguards	− 0.071***	− 0.071**	− 0.071**
5 $\times$ Care home vanguards	− 0.052***	− 0.054	− 0.054
6 $\times$ Care home vanguards	− 0.044***	− 0.046	− 0.046
7 $\times$ Care home vanguards	− 0.030	− 0.031	− 0.031
8 $\times$ Care home vanguards	− 0.057**	− 0.059*	− 0.059*
9 $\times$ Care home vanguards	− 0.062***	− 0.064*	− 0.064*
10 $\times$ Care home vanguards	− 0.083***	− 0.084**	− 0.084**
11 $\times$ Care home vanguards	− 0.084***	− 0.084**	− 0.084**
12 $\times$ Care home vanguards	− 0.105***	− 0.105***	− 0.105***
13 $\times$ Care home vanguards	− 0.081***	− 0.081**	− 0.081**
14 $\times$ Care home vanguards	− 0.091***	− 0.090***	− 0.090***
15 $\times$ Care home vanguards	− 0.082***	− 0.082**	− 0.082**
16 $\times$ Care home vanguards	− 0.061***	− 0.062*	− 0.062*
17 $\times$ Care home vanguards	− 0.067***	− 0.069**	− 0.069**
18 $\times$ Care home vanguards	− 0.067**	− 0.069**	− 0.069**

This table shows difference-in-differences estimates of emergency admissions rates in the 22 months post-Vanguard for integrated care and care home initiatives compared to non-Vanguard sites and the pre-Vanguard period. The last two columns show the same estimations but estimated at group level. These are estimated for 3 groups over 81 months and therefore use 243 observations. Base period is the quarter immediately preceding the onset of the Vanguard period

*$$p<0.10$$, **$$p< 0.015$$, ***$$p< 0.01$$

## Appendix 2: Effects of confounding policies

An integrated care initiative that closely preceded Vanguard, the Integrated care and support Pioneer programme ran from 2013 to 2018. Thus the programme overlapped in time and in some instances in place as well with the Vanguard programme. Morciano et al. [16] previously compare the effect of Pioneer and Vanguard programme. They found that emergency admission rates grew less overtime in sites that participated in both programmes.

In our sample there were 66 sites (including Vanguards and non-Vanguards) that were also exposed to the pioneer programme. To the extent that those participating in pioneer programmes may have led to stronger legacy effects, we re-estimate our main model (Eq. 1) for those sites which never participated in the Pioneer programme. In Table 4, we report the results from our main sample and compare this with our ‘no pioneers’ sample. In the ‘no pioneers’ sample there are 13 population-based Vanguards and 3 care home Vanguards. For population-based vanguards, the effects earlier seen in the last few quarters before the end of the programme (first column) are no longer significant. But the legacy effects are still evident (strong effects are evident particularly in quarters 15 and 18).

For care home sites, the effects from the ‘no pioneers’ sample appear to follow a similar pattern as the main sample. We do see legacy effects that are significant until the last two quarters of the sample. The magnitude of the effect appears slightly larger during the programme and starts to become smaller after the end of the programme, and become non-significant in the last two quarters.

Our analysis suggests that possibly due to a head start in integrated care initiatives, pioneer sites may have contributed to more stable and longer lasting effects. However, our evidence also indicates that the non-pioneer sites had detectable effects (particularly for care home Vanguards) that continued up to four quarters after the end of the programme.

**Table 4:** Difference-in-differences estimates: Vanguards versus Pioneers

	All	No pioneers
*Quarter* $\times$ *Vanguard* ($j=1$)		
− 8 $\times$ Population-based vanguards	− 0.023	− 0.027
− 7 $\times$ Population-based vanguards	− 0.023	− 0.033
− 6 $\times$ Population-based vanguards	− 0.026	− 0.009
− 5 $\times$ Population-based vanguards	− 0.035*	− 0.027
− 4 $\times$ Population-based vanguards	− 0.010	− 0.003
− 3 $\times$ Population-based vanguards	− 0.019	− 0.025
− 2 $\times$ Population-based vanguards	0.010	0.015
0 $\times$ Population-based vanguards	− 0.012	− 0.005
1 $\times$ Population-based vanguards	− 0.018	− 0.012
2 $\times$ Population-based vanguards	− 0.037	− 0.018
3 $\times$ Population-based vanguards	− 0.008	− 0.003
4 $\times$ Population-based vanguards	− 0.005	0.007
5 $\times$ Population-based vanguards	0.003	0.016
6 $\times$ Population-based vanguards	− 0.007	− 0.007
7 $\times$ Population-based vanguards	− 0.009	− 0.012
8 $\times$ Population-based vanguards	− 0.032	− 0.025
9 $\times$ Population-based vanguards	− 0.046**	− 0.042
10 $\times$ Population-based vanguards	− 0.040	− 0.029
11 $\times$ Population-based vanguards	− 0.047**	− 0.041
12 $\times$ Population-based vanguards	− 0.043*	− 0.027
13 $\times$ Population-based vanguards	− 0.063***	− 0.051*
14 $\times$ Population-based vanguards	− 0.046*	− 0.028
15 $\times$ Population-based vanguards	− 0.064***	− 0.060**
16 $\times$ Population-based vanguards	− 0.071***	− 0.058*
17 $\times$ Population-based vanguards	− 0.041	− 0.033
18 $\times$ Population-based vanguards	− 0.052**	− 0.068**
*Quarter* $\times$ *Vanguard* ($j=2$)		
− 8 $\times$ Care home vanguards	− 0.028	− 0.062*
− 7 $\times$ Care home vanguards	− 0.035	− 0.042
− 6 $\times$ Care home vanguards	− 0.028	− 0.038
− 5 $\times$ Care home vanguards	− 0.015	− 0.022
− 4 $\times$ Care home vanguards	0.004	− 0.002
− 3 $\times$ Care home vanguards	0.019	0.036
− 2 $\times$ Care home vanguards	0.020	0.018
0 $\times$ Care home vanguards	− 0.010	0.001
1 $\times$ Care home vanguards	− 0.022	− 0.013
2 $\times$ Care home vanguards	− 0.038**	− 0.046*
3 $\times$ Care home vanguards	− 0.048***	− 0.055**
4 $\times$ Care home vanguards	− 0.071***	− 0.073***
5 $\times$ Care home vanguards	− 0.052***	− 0.049*
6 $\times$ Care home vanguards	− 0.044***	− 0.053**
7 $\times$ Care home vanguards	− 0.030	− 0.047*
8 $\times$ Care home vanguards	− 0.057**	− 0.052
9 $\times$ Care home vanguards	− 0.062***	− 0.051
10 $\times$ Care home vanguards	− 0.083***	− 0.084***
11 $\times$ Care home vanguards	− 0.084***	− 0.083**
12 $\times$ Care home vanguards	− 0.105***	− 0.090***
13 $\times$ Care home vanguards	− 0.081***	− 0.064*
14 $\times$ Care home vanguards	− 0.091***	− 0.076**
15 $\times$ Care home vanguards	− 0.082***	− 0.078**
16 $\times$ Care home vanguards	− 0.061***	− 0.045*
17 $\times$ Care home vanguards	− 0.067***	− 0.041
18 $\times$ Care home vanguards	− 0.067**	− 0.050

This table shows difference-in-differences estimates of emergency admissions rates for main sample and in a reduced sample where none of the sites were participants in the pioneer programme. From all Vanguard and non-Vanguard sites, 66 sites were concurrently pioneers, so the reduced sample comes from 138 sites(13 PACS/MCP, 3 ECH, 122 non-Vanguard)

*$$p<0.10$$, **$$p< 0.015$$, ***$$p< 0.01$$

## Appendix 3: Clustering standard errors

Recent econometric research has debated upon whether and when clustering of standard errors seems relevant. Abadie et al. [27] suggest that the decision to cluster should be based on treatment assignment or data sampling. In our study, we have total population data but treatment assignment happens at site level (i.e., several sites applied to be Vanguards and 50 were selected). In this case, we have few treated clusters—24 population based sites and 5 care home sites. When there are few clusters, Abadie et al. [27] suggest that clustering leads to conservative estimates and Roth et al. [28] indicate that this may lead to under-rejection of the null of parallel trends.

We cluster at site level and report the estimates in Table 5 to demonstrate where the problem occurs in contrast to robust standard errors.

**Table 5:** Difference-in-differences estimates: robust versus clustered errors

	Robust	Cluster ID
*Quarter* $\times$ *Vanguard* ($j=1$)		
− 8 $\times$ Population-based vanguards	− 0.023	− 0.023
− 7 $\times$ Population-based vanguards	− 0.023	− 0.023
− 6 $\times$ Population-based vanguards	− 0.026	− 0.026
− 5 $\times$ Population-based vanguards	− 0.035*	− 0.035**
− 4 $\times$ Population-based vanguards	− 0.010	− 0.010
− 3 $\times$ Population-based vanguards	− 0.019	− 0.019*
− 2 $\times$ Population-based vanguards	0.010	0.010
0 $\times$ Population-based vanguards	− 0.012	− 0.012
1 $\times$ Population-based vanguards	− 0.018	− 0.018
2 $\times$ Population-based vanguards	− 0.037	− 0.037*
3 $\times$ Population-based vanguards	− 0.008	− 0.008
4 $\times$ Population-based vanguards	− 0.005	− 0.005
5 $\times$ Population-based vanguards	0.003	0.003
6 $\times$ Population-based vanguards	− 0.007	− 0.007
7 $\times$ Population-based vanguards	− 0.009	− 0.009
8 $\times$ Population-based vanguards	− 0.032	− 0.032**
9 $\times$ Population-based vanguards	− 0.046**	− 0.046***
10 $\times$ Population-based vanguards	− 0.040	− 0.040*
11 $\times$ Population-based vanguards	− 0.047**	− 0.047*
12 $\times$ Population-based vanguards	− 0.043*	− 0.043*
13 $\times$ Population-based vanguards	− 0.063***	− 0.063***
14 $\times$ Population-based vanguards	− 0.046*	− 0.046
15 $\times$ Population-based vanguards	− 0.064***	− 0.064**
16 $\times$ Population-based vanguards	− 0.071***	− 0.071***
17 $\times$ Population-based vanguards	− 0.041	− 0.041
18 $\times$ Population-based vanguards	− 0.052**	− 0.052*
*Quarter* $\times$ *Vanguard* ($j=2$)		
− 8 $\times$ Care home vanguards	− 0.028	− 0.028
− 7 $\times$ Care home vanguards	− 0.035	− 0.035**
− 6 $\times$ Care home vanguards	− 0.028	− 0.028
− 5 $\times$ Care home vanguards	− 0.015	− 0.015
− 4 $\times$ Care home vanguards	0.004	0.004
− 3 $\times$ Care home vanguards	0.019	0.019
− 2 $\times$ Care home vanguards	0.020	0.020*
0 $\times$ Care home vanguards	− 0.010	− 0.010
1 $\times$ Care home vanguards	− 0.022	− 0.022*
2 $\times$ Care home vanguards	− 0.038**	− 0.038**
3 $\times$ Care home vanguards	− 0.048***	− 0.048
4 $\times$ Care home vanguards	− 0.071***	− 0.071***
5 $\times$ Care home vanguards	− 0.052***	− 0.052*
6 $\times$ Care home vanguards	− 0.044***	− 0.044*
7 $\times$ Care home vanguards	− 0.030	− 0.030
8 $\times$ Care home vanguards	− 0.057**	− 0.057
9 $\times$ Care home vanguards	− 0.062***	− 0.062
10 $\times$ Care home vanguards	− 0.083***	− 0.083*
11 $\times$ Care home vanguards	− 0.084***	− 0.084*
12 $\times$ Care home vanguards	− 0.105***	− 0.105**
13 $\times$ Care home vanguards	− 0.081***	− 0.081
14 $\times$ Care home vanguards	− 0.091***	− 0.091**
15 $\times$ Care home vanguards	− 0.082***	− 0.082*
16 $\times$ Care home vanguards	− 0.061***	− 0.061*
17 $\times$ Care home vanguards	− 0.067***	− 0.067***
18 $\times$ Care home vanguards	− 0.067**	− 0.067**

This table shows difference-in-differences estimates of emergency admissions rates for robust standard errors and those clustered at unit level. For the case of clustered standard errors, the pre-trend tests are jointly significant at 1%

*$$p<0.10$$, **$$p< 0.015$$, ***$$p< 0.01$$

## Appendix 4: Parallel trends in the pre-Vanguard period

To correctly establish the causal effect of the programme, a crucial assumption is that the trends between treatment and control groups would have been parallel in the post-treatment period in the absence of the intervention. However, since that is untestable, the alternative is to test whether the trends were parallel in the pre-treatment period. In Fig. 3, we observed that the interaction terms estimated for the pre-treatment period (quarter $<0$) were not significant for both, population-based and care home Vanguards.^16^

As a robustness check, we drop the initial quarter (April–July 2013) instead of the 1st quarter before treatment, and report our estimates from the event history analysis shown in Fig. 6. We do not find any significant difference at 5% level in the estimated parameters for the two treated groups in the pre-treatment period.

Earlier in the paper (in “Results”), we noted that the *p* value for parallel trends test for care home vanguards is marginal. We thus follow Rambachan and Roth [32] to show the extent to which our results are robust to violations of parallel trends in the post-Vanguard period. There may be cause for concern if there are confounders in the form of other trends in the health and/or social care sector that systematically effect one group more than the other (for example-trends in funding of social care sector may affect care home sites independently of the Vanguard programme). We carry out a sensitivity check which allows us to impose smoothness restrictions, i.e. bounds to the extent to which the slope of the difference in trends can vary across consecutive periods. We compute this for the first post-Vanguard period (quarter $=12$) for care home Vanguards which is shown in Fig. 7. From the plot, it can be observed that the significant results for care home Vanguards are robust until $M\approx 0.002$. It is observed that the confidence intervals when allowing for linear violations of parallel trends ($M\approx 0$) are similar to OLS, but they become wider when we allow for larger deviations from linearity. The overall picture implies that our robust results allow for deviations from non-linearity of no more than 0.002 across consecutive periods.

To put these results in context, we go back to our possible confounders to underlying linear trends, such as the effect of social care funding. Earlier studies that have considered the effect of cuts to long-term care funding on hospital use among the elderly in England, such as Crawford et al. [40], found no significant effects upon mean emergency admissions, but did find significant effects for short stay emergency admissions (less than 3 days). Their estimates suggest that a $\pounds$100 decrease in per capita long-term care spending led to an increase of 0.002 emergency admissions that last less than 1 day and an increase of 0.004 emergency admissions that last 3 days or less. If we interpret this as the effect of changes in underlying trends, then our sensitivity checks suggest that the core results are robust to the slope of the differential trend changing by the equivalent of the full effect of the emergency admissions less than 1 day and up to 50% of the effect of emergency admission spells that last 3 days.

## Footnotes

1 Similar underlying mechanisms have been studied in contexts such as, poverty alleviation and child health [19, 20] and quality of health care delivery [5, 6].

2 These were 8 Urgent and Emergency Care Vanguards aimed at improving coordination and reducing pressure on Accident and Emergency departments. As well as 13 Acute Care Collaborations working towards linking hospitals and improving both clinical and financial viability.

3 We received data up to March 2020, but we drop the last month for the possibility of any discrepancies. Moreover, with pre-pandemic data we avoid any bias in our estimation of the effect of programme discontinuation.

4 Where a CCG has a Vanguard site along with some other practices which are not exposed to the programme, the same CCG has two entries. However, they have variation in population structure corresponding to the participating vs non-participating regions.

5 The raw data included 184 sites that were coded as non-Vanguards. We dropped 5 sites which had missing population values and another 4 sites that had unbalanced number of observations. Note these sites show group of practices, but we do not observe data at individual care home or practice level.

6 The advantage of this approach over synthetic measures of inequality computed from various metrics of the underlying distribution (based on, e.g. associated interquartile ranges, standard deviation, Gini coefficient, etc.) lies in the possibility of a graphical assessment on whether convergence occurred mainly because of changes among the best (25th percentile) or the worst (75th percentile) performing sites.

7 We may be able to assume this condition to hold if pre-determined variables are unlikely to be affected by treatment [29]. In our context, this implies that if variables such as area level-population structure, level of deprivation, disease prevalence are unaffected by treatment, then rank similarity may not be violated.

8 For example, Frandsen and Lefgren [30] show this in the context of class size assignment experiment on student test scores, as distribution of students with lower scores are not invariant to predetermined family income levels. Therefore, treatment (assignment to smaller classes) may benefit struggling students from low-income backgrounds more than those from high income backgrounds.

9 The null hypothesis of parallel trends was not rejected at 5% statistical levels for the Vanguard population-based (*p*-value = 0.94) and care home (*p*-value = 0.07) sites nor by the event history analysis reported in Fig. 3. It should be noted though that the parallel trends test for care home Vanguards may marginally (at 10%) be rejected. To check the extent to which our results are sensitive to violations of post-treatment parallel trends, we follow the method proposed by Rambachan and Roth [32] and discuss this in “Appendix 4: Parallel trends in the pre-Vanguard period”. As an additional robustness check, we exclude the initial quarter (April–July 2013) and report our event history results in Fig. 6 in “Appendix 4: Parallel trends in the pre-Vanguard period”.

10 The full list of results from this estimation are presented in column 1, Table 3 in “Appendix 1: Testing aggregation bias”. We also contrast these results with those obtained from aggregating data at Vanguard group level and discuss the resultant aggregation bias.

11 The effect is insignificant at the end of year 2 and beginning of year 3 (quarters 7, 8 and 9).

12 The effect is insignificant for quarter 10 in year 3. In the post-Vanguard period, the effect is significant for all, except quarters 14 and 17.

13 As mentioned before, absence of any movements would be evidence of rank preservation in it’s strictest sense.

14 An alternative is to consider funding allocation across Vanguard sites. However, funding itself was endogenous to the performance of any single Vanguard site, with sites deemed not to be performing at the end of year one denied funding for year two [11]. Qualitative research confirms that additional funding was considered crucial and many initiatives were downgraded/withdrawn when funding was withdrawn [34].

15 Previous qualitative research [11] has also indicated that local support structures that were put in place had valuable effects in terms of enhancing trust and enthusiasm among participating sites. However, given the unmeasurable nature of these inputs, we were not able to quantify their impact upon outcomes for Vanguard sites. Nevertheless, these are likely to have reduced between-group variation while being more beneficial to the worst performing sites, in line with what we have found with the quantile regression analysis.
